# Correlation of Perfusion Index Change and Analgesic Efficacy in Transforaminal Block for Lumbosacral Radicular Pain

**DOI:** 10.3390/jcm8010051

**Published:** 2019-01-07

**Authors:** Jin Young Lee, Eung Don Kim, Yoo Na Kim, Ji Seob Kim, Woo Seog Sim, Hae Jin Lee, Hyun Joon Park, Hue Jung Park

**Affiliations:** 1 Department of Anesthesiology and Pain Medicine, Samsung Medical Center, Sungkyunkwan University, School of Medicine, Seoul 06351, Korea; l7035@hanmail.net (J.Y.L.); wooseog.sim@samsung.com (W.S.S.); 2 Department of Anesthesiology and Pain Medicine, Daejeon St. Mary’s hospital, College of Medicine, The Catholic University of Korea, Seoul 34943, Korea; ehs99@catholic.ac.kr; 3 Department of Anesthesiology and Pain Medicine, Seoul St. Mary’s hospital, College of Medicine, The Catholic University of Korea, Seoul 06591, Korea; yoonayaaa@gmail.com (Y.N.K.); demiandew@naver.com (J.S.K.); lehaji@catholic.ac.kr (H.J.L.); jkhp110@naver.com (H.J.P.)

**Keywords:** change ratio, perfusion index, radicular pain, transforaminal block

## Abstract

Transforaminal epidural injection is used to treat radicular pain. However, there is no objective method of assessing pain relief following transforaminal injection. Perfusion index is a metric for monitoring peripheral perfusion status. This study evaluates the correlation between perfusion index change and analgesic efficacy in transforaminal blocks for lumbosacral radicular pain. We retrospectively analyzed data of 100 patients receiving transforaminal block for lumbosacral radicular pain. We assessed perfusion index before treatment and at 5, 15, and 30 min following the block. We defined responders (group R) and non-responders (group N) as those with ≥50% and <50% pain reduction, respectively, 30 min following block. Clinical data and perfusion index of the groups were analyzed. Ninety-two patients were examined, of whom 57 (61.9%) and 35 (38.0%) patients reported ≥50% and <50% pain reduction, respectively. Group R had a significantly higher perfusion index change ratio 5 min following the block (*p* = 0.029). A perfusion index change ratio of ≥0.27 was observed in group R (sensitivity, 75.4%; specificity, 51.4%; AUC (area under the curve), 0.636; *p* = 0.032). A perfusion index change ratio of ≥0.27 at 5 min after block is associated with, but does not predict improvement in, pain levels following lumbosacral transforaminal block.

## 1. Introduction

Epidural injection of local anesthetics and steroids is one of the most common methods of managing chronic low back pain and radicular pain [1,2]. Hyaluronidase is added to the epidural injectate, which has been known to reduce tissue swelling caused by fibrin deposits and chronic inflammation [3,4]. The transforaminal approach, one of several approaches, can deliver a small volume of injectate close to the site of pathology, presumably into an inflamed nerve root, to reduce inflammation and ischemia [1,5,6,7,8]. However, there is no objective metric to assess pain relief following injection. Commonly used screening tools and pain questionnaires include patient responses which may be affected by various factors such as comorbid conditions and/or psychosocial causes [9,10]. Early identification of pain status, however, is crucial in developing further treatment plans. Perfusion index (PI) is a quantitative value of a photoplethysmography waveform that reflects real-time changes in peripheral blood flow at the site being monitored [11]. PI changes reflect changes in the peripheral vascular beds controlled by the sympathetic nervous system [12]. Tapar and colleagues [13] evaluated the efficacy of PI as a parameter for assessing postoperative pain and response to analgesics. However, the use of PI has not yet been described in chronic back pain treatment. This study was performed to evaluate the correlation between change in PI and analgesic efficacy in patients undergoing transforaminal block.

## 2. Materials and Methods

### 2.1. Patients

We retrospectively reviewed the electronic medical records of 100 patients with lumbosacral radicular pain who underwent transforaminal block from April to August 2018 at two tertiary care hospitals. The patients ranged in age from 20 to 83 years. All patients had low back pain and radicular pain for more than 3 months. The inclusion criteria were as follows: (a) a primary diagnosis of low back pain radiating to the lower limbs; (b) a diagnosis of spinal stenosis, herniated nucleus pulposus, and/or a degenerative spinal disorder on a cross sectional imaging study (either CT or MRI) of the lumbosacral spine. The exclusion criteria included lumbosacral epidural injection within the previous 2 weeks and any history of lumbosacral surgery, lumbosacral neuroplasty, neoplastic diseases, peripheral vascular disease, or use of medications affecting the vascular system. The lesion level for transforaminal injection was determined based on clinical manifestations, physical examination, and review of imaging studies. Lesion severity was categorized as one of three different degree levels (mild, moderate, severe) by reviewing imaging data. This study was approved by our departmental ethics committee (ref: SMC 2018-08-178) and registered with CRIS (Clinical Research Information Service of the Korea National Institute of Health, http://cris.nih.go.kr/cris/index.jsp, ref: KCT0003318).

### 2.2. Intervention

All procedures were performed under fluoroscopic guidance and standardized. Patients were placed in the prone position, and anteroposterior and lateral view images were obtained with a C-arm (OEC series 9800, General Electric, Boston, MA, USA) to ensure proper site of entry. Following aseptic preparation and application of 1% lidocaine, a 23-gauge Tuohy needle (Tae-Chang Industrial Co., Seoul, Korea) was inserted into the skin surface over the upper quadrant of the target foramen. Aspirations to assess for the presence of blood or cerebrospinal fluid were routinely performed. When negative for aspirate, 0.5–2 mL of contrast medium (Omnipaque^®^, 300 mgI·mL^−1^, GE Healthcare, Little Chalfont, Buckinghamshire, UK) was injected to confirm that the needle tip was appropriately placed in the epidural space. After confirming that the contrast had spread throughout the epidural space, a total volume of 5 mL (containing 0.4% lidocaine, dexamethasone, hyaluronidase 750 IU, and normal saline) was infused. Following the procedure, patients were observed for any adverse effects. PI was monitored using pulse oximetry (Root^®^, Masimo Corporation, Irvine, CA, USA) on the toe of the affected limb. We assessed PI prior to treatment (T0), and at 5 (T5), 15 (T15), and 30 (T30) min following transforaminal injection. Temperature was assessed using a touch thermometer (IntelliVue MP70 patient monitor, Philips Healthcare, Best, Netherlands) on the dorsum of the foot of the affected limb. Room temperature was maintained at 23–25 °C. Pain was scored using a numerical rate scale (NRS, ranging from 0 = no pain to 10 = absolutely intolerable pain) and cold sensation of the affected limb (0 = no cold, 1 = mild cold, 2 = moderate cold, 3 = severe cold) was recorded at T10 and T30. PI, temperature, pain severity, and cold sensation at T0 were recorded after 5 min of bed rest and before skin infiltration with 1% lidocaine.

### 2.3. Statistical Analysis

All data were analyzed using SAS 9.4 (SAS Institute, Cary, NC, USA). Data are expressed as the mean ± standard deviation (SD) or number (proportion), as appropriate. Demographic data for the two groups were compared using a Chi-square test, T-test or Fisher’s exact test. To minimize individual variance in PI absolute values, we calculated PI change ratios (PI at each time point—PI at T0/PI at T0) and temperature changes (temperature at each time point—temperature at T0) at T5, T15, and T30. The PI change ratios, temperature change, pain severity, and cold sensation over time were compared using the Wilcoxon rank sum test. In each group, the differences in PI change ratios over time were compared using a generalized estimating equations analysis. A receiver operating characteristic (ROC) curve was constructed to investigate the cut-off PI change ratio at T5 to correctly predict analgesic efficacy at the maximum area under the curve (AUC), which ranged from 0.5 to 1.0. A *p*-value less than 0.05 was considered statistically significant. 

## 3. Results

**Figure 1:**
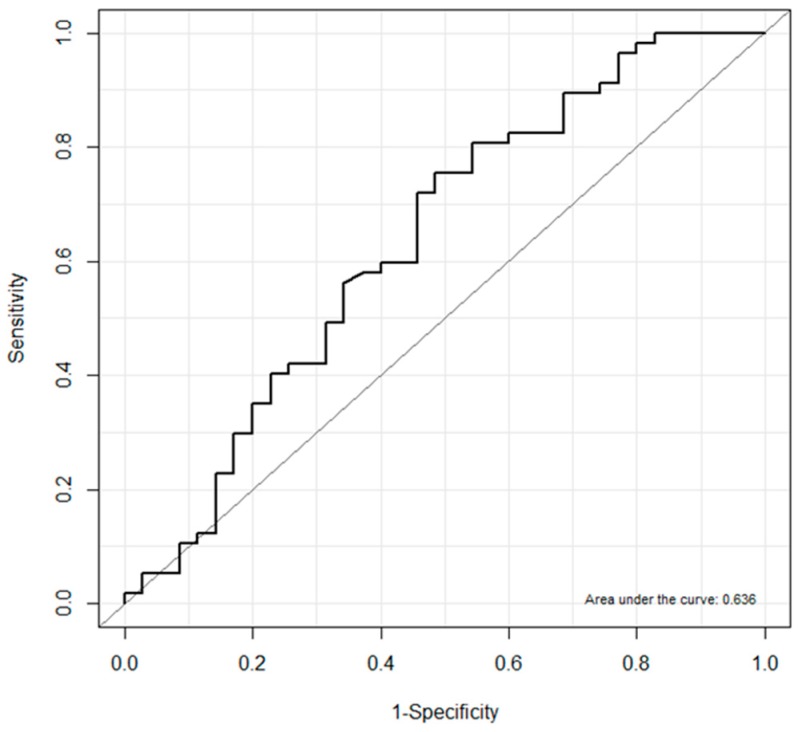
Receiver operating characteristic curve of the perfusion index change ratio at 5 min following block for predicting analgesic efficacy in group R (*n* = 57). The optimal cut-off point for the perfusion index change ratio was ≥0.27 with an area under the curve of 0.636.

Of the 100 patients assessed for eligibility, eight were excluded due to insufficient medical records (*n* = 6) or failed transforaminal injection due to epidural venogram (*n* = 2). Thus, a total of 92 patients were analyzed. We defined responders, or group R, as patients who showed a reduction of ≥50% on the numeric rate scale for pain, 30 min following the block, and non-responders, or group N, as those who showed a reduction of less than 50%. Demographic and clinical data are summarized in Table 1. Age, sex, body mass index, diagnosis, duration of pain, lesion level, lesion severity, injection level, or injection side did not differ between the two groups (Table 1). The PI change ratio and temperature change were presented as mean ± SD (Table 2). Group R showed a significantly higher PI change ratio at T5 compared with group N (*p* = 0.029). There were no significant differences in PI change ratios over time in group R (*p* = 0.351) or in group N (*p* = 0.654). The ROC curve of PI change ratios at T5 is shown in Figure 1. PI change ratio ≥0.27 was observed in group R (sensitivity, 75.4%; specificity, 51.4%; AUC, 0.636; *p* = 0.032). The change in temperature was not different between the two groups (Table 2). Cold sensation was also not different between the two groups (Table 3). None of the cases showed any evidence of dural puncture or neurological complications.

## 4. Discussion

Lumbosacral radicular pain is caused by irritation or compression of the affected nerve root [14]. It is caused by a direct mass effect on the nerve root as well as by chemically mediated inflammatory reactions [15]. Previous studies have reported evidence of a potential connection between chronic low back pain and impaired perfusion [16]. Lumbar ischemia can lead to several different outcomes depending on the development of collateral circulation of the lumbar feeding artery, such as vertebral bone ischemia presenting as constant dull pain and nerve root ischemia presenting as radicular pain [16]. Transforaminal block is a valid procedure for the diagnosis and treatment of lumbosacral radicular pain [17]. When considering lumbar surgery, changes in pain or disability status following transforaminal injection are used to categorize surgical candidates [17]. However, patients’ responses may be subjective. PI has recently received greater attention in the fields of anesthesia and analgesia [13,18,19]. PI is a ratio between the pulsatile and non-pulsatile signals, reflecting peripheral perfusion [20,21]. When the sympathetic nervous system is activated, PI may decrease due to increased vasomotor tone and contraction of peripheral blood vessels [18]. Surgical or other noxious stimuli and cold stress cause vasoconstriction, leading to changes in PI [12,22]. Mowafi and colleagues [22] showed that PI is a reliable measure for the intravascular injection of epinephrine during epidural anesthesia. Some reports have described the use of PI in various conditions, namely, for pain assessment in critically ill patients, at the onset of stellate ganglion block, in lumbar and thoracic sympathectomy, or for prediction of the success of brachial plexus or sciatic nerve blocks [21,23,24,25,26].

In this study, we found that PI change ratios at T5 in group R were significantly higher compared with those in group N. We also found that a PI change ratio of ≥0.27 can be a useful marker of improvement of pain. The sensitivity of 75.4%, specificity of 51.4%, and AUC of 0.636 in the current study were relatively lower than these parameters in previous studies [19,22,24,25]. We suspect that this was because our study was based on analgesia, not anesthesia, which requires deep sedation and muscle relaxation. This could explain the large standard deviation in PI change ratio at T30, related to artifacts produced by movement during the monitoring period. PI measurements are quite sensitive to patients’ movement and that of the probe/tissue, which may cause rapid fluctuations in PI values [12]. The rapid fluctuation and sensitivity of PI are its weakness as well as strength in the clinical field. To compensate for this limitation, PI monitoring in the awake patient should be done after ensuring stability of position, temperature, and emotional status after sufficient rest. In addition to the physician’s judgement and the circumstances of the individual patients, PI change ratios can be one of the indicators in planning further interventions. Even though we were unable to identify the cause of the higher PI change ratios following the block in group R, we suspect that it was due to pain relief rather than improved spinal perfusion since temperature change and cold sensation did not differ between the groups. Based on this result, we can also rule out the possibility that PI changes owing to lumbar sympathetic spread by transforaminal injection of a volume of 5 mL. 

This study had several limitations. First, we did not measure the PI of the contralateral limb. Second, we categorized patients using only a numerical rate scale. In addition, we did not include an evaluation of functional disability. Third, the follow-up period of 30 min was too short to sufficiently evaluate block efficacy. Joswig and colleagues [27] reported that it is impossible to reliably predict long-term treatment responses based on short-term pain relief. Finally, each patient took various analgesics or underwent other interdisciplinary management protocols that could have affected the severity of the pain after block.

## 5. Conclusions

This study shows that PI change ratios are associated with successful pain relief following lumbosacral transforaminal block, with a PI change ratio of ≥0.27 at T5 reflecting an improvement in pain. PI change ratios can provide a simple and easy way of monitoring overall changes in pain levels, but they cannot predict improvement in pain owing to the low AUC. Future prospective randomized studies are needed to determine whether PI change ratios can provide superior diagnostic value in the long-term period after injection and for the treatment of other forms of chronic pain.

## Figures and Tables

**Table 1:** Patient demographic and clinical data.

	All Patients (*n* = 92)	Group R (*n* = 57)	Group N (*n* = 35)	*p*-Value
Age (year)	62.1 ± 13.8	63.1 ± 13.1	60.4 ± 14.9	0.385
Sex (M/F)	42/50	29/28	13/22	0.285
Body mass index (kg/m^2^)	24.8 ± 4.6	24.7 ± 3.7	24.6 ± 5.8	0.895
Diagnosis				0.872
Spinal stenosis	56 (60.9%)	35 (61.4%)	21 (60.0%)	
HNP	32 (34.8%)	20 (35.1%)	12 (34.3%)	
Others	4 (4.3%)	2 (3.5%)	2 (5.7%)	
Duration of pain				0.812
<3 months	26 (28.3%)	17 (29.8%)	9 (25.7%)	
3–12 months	20 (21.7%)	13 (22.8%)	7 (20.0%)	
>12 months	46 (50.0%)	27 (47.4%)	19 (54.3%)	
Lesion level				0.702
L2–3	3 (3.3%)	3 (5.3%)	0 (0.0%)	
L3–4	7 (7.6%)	4 (7.0%)	3 (8.6%)	
L4–5	60 (65.2%)	37 (64.9%)	23 (65.7%)	
L5–S1	22 (23.9%)	13 (22.8%)	9 (25.7%)	
Lesion severity				0.434
Mild	25 (27.2%)	17 (29.8%)	8 (22.9%)	
Moderate	47 (51.1%)	30 (52.6%)	17 (48.6%)	
Severe	20 (21.7%)	10 (17.5%)	10 (28.6%)	
Injection level (1 level)				0.730
L2	0 (0.0%)	0 (0.0%)	0 (0.0%)	
L3	1 (1.1%)	0 (0.0%)	1 (2.9%)	
L4	24 (26.1%)	14 (24.6%)	10 (28.6%)	
L5	26 (28.3%)	16 (28.1%)	10 (28.6%)	
S1	8 (8.7%)	4 (7.0%)	4 (11.4%)	
Injection level (2 levels)				0.606
L2, 3	2 (2.2%)	2 (3.5%)	0 (0.0%)	
L4, 5	27 (29.3%)	19 (33.3%)	8 (22.9%)	
L5, S1	4 (4.3%)	2 (3.5%)	2 (5.7%)	
Injection side				
Left/Right	45/47	28/29	17/18	1.000

All data are presented as the mean ± standard deviation (SD) or the number of patients (%). M/F: male/female, HNP: herniated nucleus pulposus, Group R: patients who showed a reduction of ≥50% on the numeric rate scale for pain, 30 min following the block, Group N: patients who showed a reduction of less than 50%; *p*-value < 0.05 was considered statistically significant.

**Table 2:** Perfusion index change ratio and temperature change over time.

	Group R (*n* = 57)	Group N (*n* = 35)	*p*-Value
PI change ratio			
T5	1.67 ± 4.2 *	0.81 ± 1.6	0.029
T15	1.47 ± 2.4	0.97 ± 2.1	0.072
T30	6.15 ± 36.4	0.72 ± 1.8	0.104
Temperature change			
T5	0.05 ± 0.3	0.01 ± 0.3	0.391
T15	−0.02 ± 0.4	−0.01 ± 0.3	0.824
T30	−0.08 ± 0.4	−0.09 ± 0.4	0.958

PI: perfusion index, T0: before treatment, T5: 5 min following block, T15: 15 min following block, T30: 30 min following block, PI change ratio (PI at each time point—PI at T0/PI at T0), Temperature change (temperature at each time point—temperature at T0), Group R: patients who showed a reduction of ≥50% on the numeric rate scale for pain, 30 min following the block, Group N: patients who showed a reduction of less than 50%; * *p*-value < 0.05 was considered statistically significant.

**Table 3:** Perfusion index change ratio and temperature change over time.

	Group R (*n* = 57)	Group N (*n* = 35)	*p*-Value
Pain severity (NRS)			
T0	5.75 ± 1.9	5.66 ± 1.7	0.711
T30	1.05 ± 1.1	4.37 ± 1.3	<0.001
Cold sensation			
T0	0.60 ± 1.0	0.46 ± 0.9	0.638
T30	0.21 ± 0.5	0.26 ± 0.6	0.725

All data are presented as the mean ± SD, NRS: numerical rate scale, T0: before treatment, T30: 30 min following block, Cold sensation (0 = no cold, 1 = mild cold, 2 = moderate cold, 3 = severe cold), Group R: patients who showed a reduction of ≥50% on the numeric rate scale for pain, 30 min following the block, Group N: patients who showed a reduction of less than 50%; *p*-value < 0.05 was considered statistically significant.
